# Evaluation of static friction, surface roughness and ion release of stainless steel and nickel-titanium orthodontic arch wires coated with titanium dioxide nanoparticles and silver nanoparticles: in vitro study

**DOI:** 10.1186/s12903-026-08518-w

**Published:** 2026-05-15

**Authors:** Amany M. Elsonny, Nehal F. Albelasy, Marwa S. Shamaa, Shaza M. Hammad

**Affiliations:** Orthodontic Department, Faculty of Dentistry, Mansoura University, Mansoura, Egypt

**Keywords:** Orthodontic arch wires, Titanium dioxide nanoparticles, Silver nanoparticles

## Abstract

**Background:**

This study aimed to assess the impact of silver and titanium dioxide nano-coatings on stainless steel and nickel titanium arch wires concerning friction, surface roughness and the release of nickel, silver, and titanium ions.

**Methods:**

A total of 168 arch wires measuring 17.8 cm in length were divided into two groups based on the type of the orthodontic arch wire material: 84 stainless steel (SS) wires and 84 nickel titanium (NiTi) wires. Each group of these wire materials was evenly divided into three subgroups according to the nano coating material: non-coated group (control), titanium dioxide nanoparticles (TiO_2_NPs) and silver nanoparticles (Ag-NPs). Static friction on a specially designed acrylic plate was measured using a universal testing machine. Surface roughness data was then gathered using a profilometer machine, and inductively coupled plasma-optical emission spectroscopy (ICP-OES) was used to determine the ion release of Ni, Ag and Ti ions. The collected data were compared using a two-way ANOVA and the Bonferroni test for repeated pairwise comparisons. In the ion release test all samples had been incubated in 10 milliliters of artificial saliva and the data was collected at intervals of after 48 h, one week and two weeks, respectively. Then compared using a three-way mixed ANOVA.

**Results:**

The nano-coating effect on arch wires static frictional resistance was statistically significantly higher in the nanocoated wires especially in nanocoated SS arch wires (*p* < .001). The surface roughness of both arch wires is significantly impacted by the nanocoating as it became lower in nanocoated arch wires especially Ag-NPs coated SS arch wires (*p* < .001). Ni ion release significantly decreased in SS arch wires coated with TiO_2_NPs in comparison to SS wires coated with Ag-NPs and to NiTi arch wires coated with Ag-NPs and TiO_2_NPs (*p* < .001).

**Conclusions:**

Ag-NPs and TiO_2_NPs coatings are suitable for decreasing static friction in arch wires and improving surface roughness. Nano coating can improve surface roughness, static friction resistance especially for SS arch wires. Ion release of Ag and Ti ions in NiTi and SS coated arch wires is in safety concerns associated with nanoparticle dimensions. Ni ion release decreased in nano-coatings so, nano-coatings slightly improved the corrosion resistance especially in SS arch wires coated with TiO_2_NPs.

**Supplementary Information:**

The online version contains supplementary material available at 10.1186/s12903-026-08518-w.

## Background

Many patients are treated with orthodontic appliances to correct malocclusion and to enhance the patient’s quality of life and cosmetic results. To keep the jaw and teeth in the right alignment, the appliance is positioned over an extended length of time [1]. In recent decades, numerous wire alloys have been created, each possessing distinct mechanical properties. This has considerably improved the adaptability of orthodontic therapy [2]. Researchers have consistently regarded stainless steel (SS) material as the benchmark for evaluating and comparing the qualities of new arch wires in the field [3]. Orthodontic arch wires which made from nickel-titanium (NiTi) alloy, are the most effective shape memory alloy [4].

Insoluble substances with a diameter of less than 100 nanometers are known as nanoparticles (NPs). In orthodontic therapy, several primary techniques have been employed to prevent microbial adhesion or demineralization of enamel. Certain NPs such as fluorohydroxyapatite, fluorapatite, hydroxyapatite, silicon dioxide (SiO_2_), titanium dioxide (TiO_2_), silver (Ag), and nanofillers are incorporated into orthodontic adhesives, acrylic resins, and arch wires [5]. Recently, significant focus has been directed towards TiO_2_NPs due to their low toxicity and photocatalytic properties [6].

Modifications to arch wire surfaces, including coatings and surface treatments, have been proposed to reduce friction and enhance clinical performance [7]. Composite NPs and oxide NPs can be coated and synthesized using sol-gel techniques. A liquid sol is usually turned into a solid gel by the sol-gel process, which is then followed by the acquisition of a dried gel. This coating process in the lab is called the sol-gel thin film coating method which is used for coating the orthodontic materials [8].

Friction between orthodontic arch wires and brackets plays a critical role in the efficiency of tooth movement during fixed orthodontic treatment as excessive frictional resistance can reduce the effective transmission of orthodontic forces prolonging the treatment duration [9]. The magnitude of static friction in orthodontic systems is influenced by multiple factors, including arch wire material, surface roughness, bracket design, ligation method, and the oral environment [10]. In addition to mechanical performance, orthodontic arch wires are continuously exposed to the oral environment, where they are subjected to moisture, temperature fluctuations, and chemical challenges from saliva and dietary components [11], these conditions may promote corrosion and metal ion release, particularly Ni ions from NiTi and SS alloys as ion release has clinical concern due to its potential cytotoxic, allergenic, and inflammatory effects [12]. The corrosion and surface roughness of arch wires, together with ion release in the oral cavity, are shown to be positively correlated [13].

Although previous studies [14–17] have investigated nanoparticle coatings on orthodontic arch wires, many of these investigations focused on individual properties such as antimicrobial activity or friction reduction and didn’t evaluate the combined effects of nanoparticle coatings on frictional resistance, surface roughness, and metal ion release in both SS and NiTi arch wires. Therefore, the present study aimed to comprehensively assess the influence of Ag-NPs and TiO_2_NPs coatings on frictional resistance, surface roughness and ion release of the nano coating ions (Ag and Ti ions) and Ni ions which is common in the SS and NiTi arch wires.

The null hypothesis of this study states that, coating SS and NiTi orthodontic arch wires with Ag-NPs and TiO_2_NPs would not considerably decrease static friction, surface roughness and ion release of Ni, Ag and Ti ions.

## Materials and methods

### Study design and ethical approval

This in vitro experimental study was approved by the dental research ethics committee of the Faculty of Dentistry at Mansoura University in Egypt (code no. A01303024 OR).

### Collection and distribution of the dataset

This in vitro study involved 168 SS and NiTi wires of 0.017 × 0.025 inches (Orthometric, Marília, Brazil). Full SS and NiTi arch wire length of 35.6 cm were used for the ion release test while 17.8 cm pieces of SS and NiTi arch wires were used for analysis of static friction and surface roughness. Furthermore, in this study, 66 Roth prescription metal brackets (Orthometric, Marília, Brazil) with a 0.022 × 0.028-inch slot size of the lower incisors were used for the friction test. The nano-coating materials of Ag-NPs and TiO_2_NPs were prepared at the National Research Center’s laser research unit in Cairo. Artificial saliva made at Mansoura Faculty of Pharmacy’s Chemical Lab was used for the ion release test.

###  Sample size calculation

Sample size was calculated by using Power Analysis and Sample Size (PASS) Software (version 15, 2017). NCSS, LLC. Kaysville, Utah, USA.

**Fig. 1:**
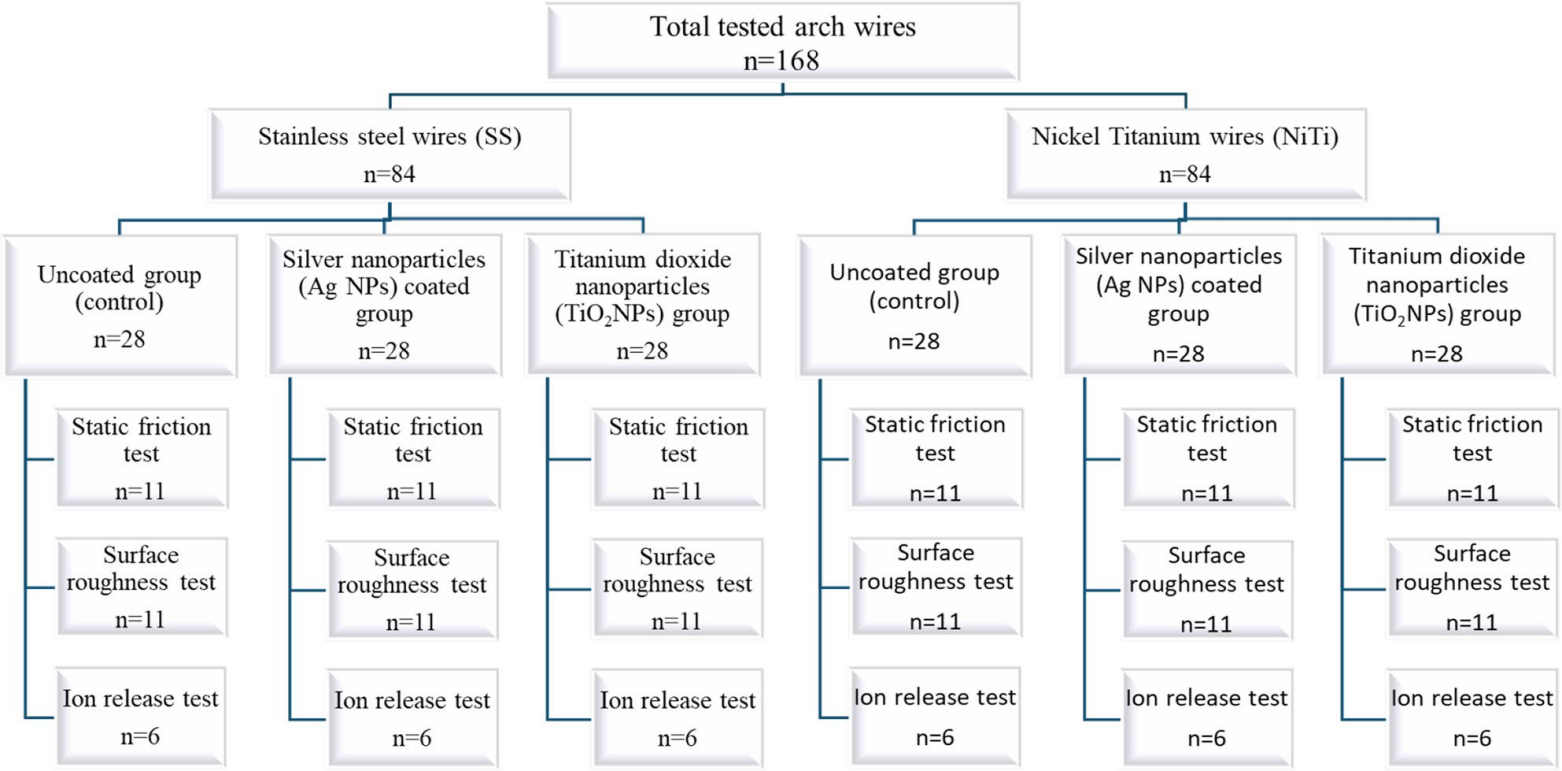
The study diagram

A total of 168 arch wires were used in this in vitro investigation. They were split up into two groups. Group 1 included 84 SS arch wires which were divided into three equal groups (28 uncoated group (control), 28 Ag-NPs coated group and 28 TiO_2_NPs coated group). Each group was subdivided into 3 subgroups (11 wires for static friction test, 11 wires for surface roughness test, and 6 wires for ion release test). Similarly group 2 contained 84 NiTi arch wires which were divided into three equal groups (28 uncoated group, 28 Ag-NPs coated group and 28 TiO_2_NPs coated group). Each group was subdivided into 3 subgroups (11 wires for static friction test, 11 wires for surface roughness test, and 6 wires for ion release test) (Fig. 1).

Sample size calculation was performed using PASS software (version 15, NCSS, Kaysville, Utah, USA). The calculation was based on detecting a significant difference in static friction values between coated and uncoated arch wires, with a significance level (α) of 0.05 and a statistical power of 80%. The estimated effect size was derived from previously published orthodontic friction studies [18, 19]. Additionally, clarification has been added regarding subgroup allocation. The distribution of samples (11 for friction testing, 11 for surface roughness, and 6 for ion release) was determined based on methodological requirements of each experimental procedure and consistency with previous in vitro orthodontic materials studies. For the primary outcome which including the static friction and surface roughness, a total of 66 subjects are required to provide 11 subjects per cell. Group sample sizes of 33 SS wires (11 Ag-NPs coated wires, 11 TiO_2_NPs coated wires and 11 uncoated wires (control)) and likely the 33 NiTi wires achieve 89% power when an F test is used to test factor A at a 5% significance level and the effect size is 0.400, achieves 82% power when an F test is used to test factor B at a 5% significance level and the effect size is 0.400, and achieves 82% power when an F test is used to test the A*B interaction at a 5% significance level and the effect size is 0.400. For Ion release test (time-dependent test) evaluation as a secondary outcome a group sample sizes of 18 SS wires (6 Ag-NPs coated wires, 6 TiO_2_NPs coated wires and 6 uncoated wires (control)) and likely the 18 NiTi wires achieve 87.64% power to reject the null hypothesis of zero effect size when the population effect size is 0.80 and the significance level (alpha) is 0.050 using a one-sided two-sample equal-variance t-test.

### Ag-NPs preparation

Ag-NPs were prepared at the National Research Center’s laser research unit in Cairo. The Nd: YAG nanosecond pulsed laser (PRII 8000 continuum laser; Electro Optics, Inc., Cairo) was used to irradiate the high-purity 99.99% Ag target with the following parameters: basic wavelength of 1064 nm, power of 4 W, pulse frequency of 10 Hz, and pulse width of 6 nm. The laser beam was focused perpendicularly onto the Ag objects using a 10-cm convex lens [20].

### TiO_2_NPs preparation

The TiO_2_NPs were synthesized at the physics research unit of the National Research Center in Cairo. mixing specified volumes of Ti isopropoxide (TTiP) and isopropanol (76.7 and 76.4 ml, respectively) into 800 ml of distilled water that had been brought to a pH of 1.5 using HNO_3_, HCl, or acetic acid. A white precipitate was generated upon the addition of TTiP. Following two days of agitation at ambient temperature, the precipitate transformed into a clear, yellowish solution. Triethylamine (TEA) was added dropwise to 100 milliliters of the Ti solution until the pH values attained 7, 9, and 11. A Teflon vessel inside a SS autoclave was subsequently filled with the resultant white precipitate suspension (150 ml, constituting 75% of the reactor volume). Two distinct temperature and duration conditions were employed for the hydrothermal treatment: 120 °C for 24 h and 150 °C for 6 h. The operating pressures at these temperatures are 475.72 kPa and 198.48 kPa, respectively. Centrifugation was employed to generate TiO_2_NPs, which were subsequently rinsed three times with distilled water. The precipitates were subsequently filtered and allowed to dry overnight at 120 °C [21].

### Technique of coating

All arch wires were sol-gel thin film dip coated. An ultrasonic cleaner at 50 Hz, 100 watts, 0.05 °C, and 36 °C cleaned the arch wires for 15 min in 95% ethyl alcohol, 15 min in 0.1 molar sodium hydroxide, and two cycles of 15-minute in distilled water. Control uncoated arch wires were cleaned twice with distilled water for 15 min. Each NPs-containing plate held 11 wires for 30 min. The Ag-NPs and TiO_2_NPs coating were perfectly aligned on the SS and NiTi arch wires. After dehydrating, they were placed in an oven at 160° for 3 min, then arch wires were stored in a dark, dry atmosphere protected from light and dust [22].

### The electron microscope

**Fig. 2:**
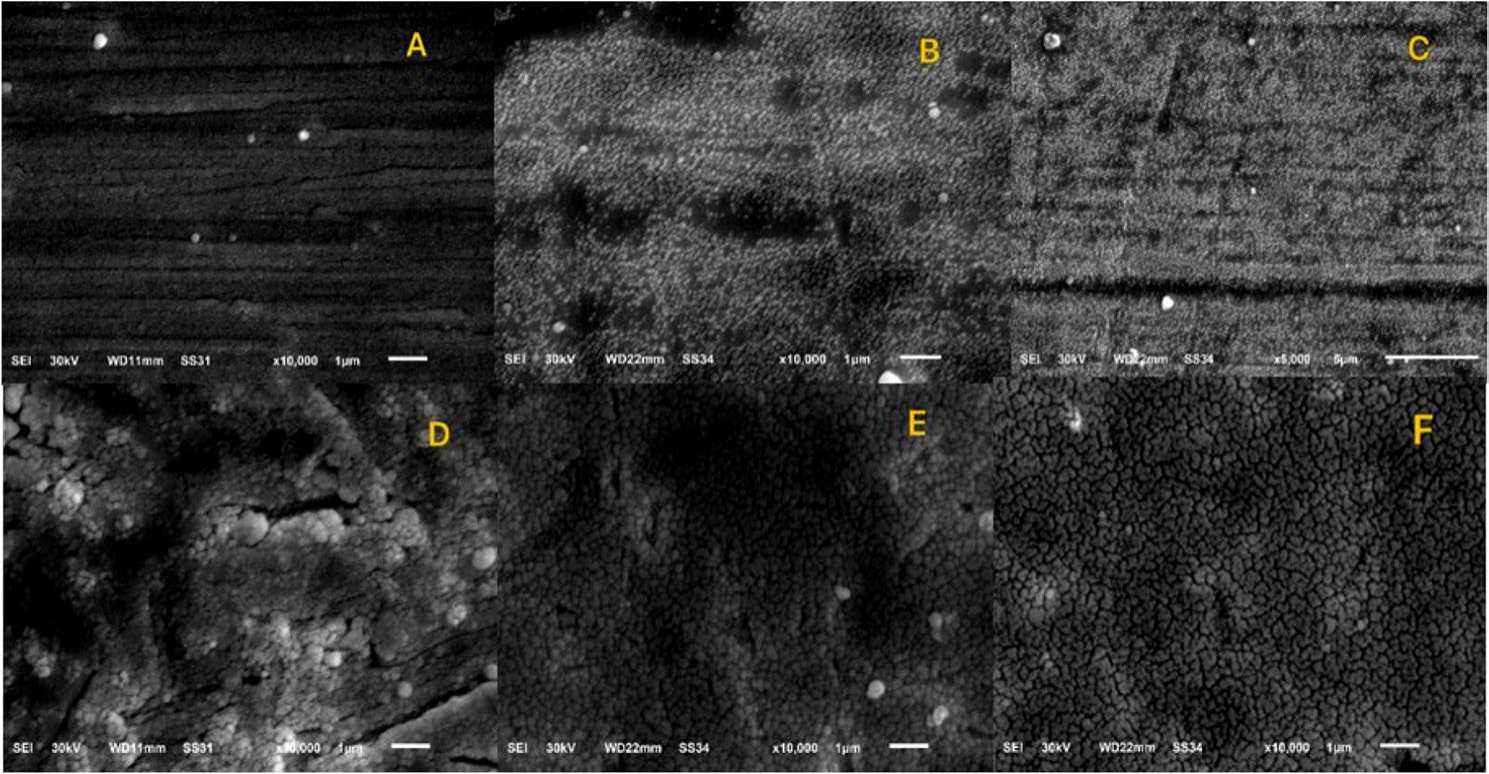
An electron microscope verifying the layers of coating on arch wire surfaces. **A** uncoated SS arch wire surface. **B** SS arch wire coated with Ag-NPs. **C** SS arch wire coated with TiO_2_NPs. **D** Noncoated NiTi arch wire surface. **E** NiTi arch wire coated with Ag-NPs. **F** NiTi arch wire coated with TiO_2_NPs

A random arch wire from each group was scanned using an electron microscope (SEM, JSM 6510, LV, Jeol, Akishima, Japan) at 1000 ×, 5000 × and 10,000 × to confirm homogeneity of nanocoating (Fig. 2). SEM analysis was performed primarily to qualitatively verify nanoparticle deposition on representative samples from each group so, a random arch wire from each group was scanned [10, 23, 24].

### Static friction test

**Fig. 3:**
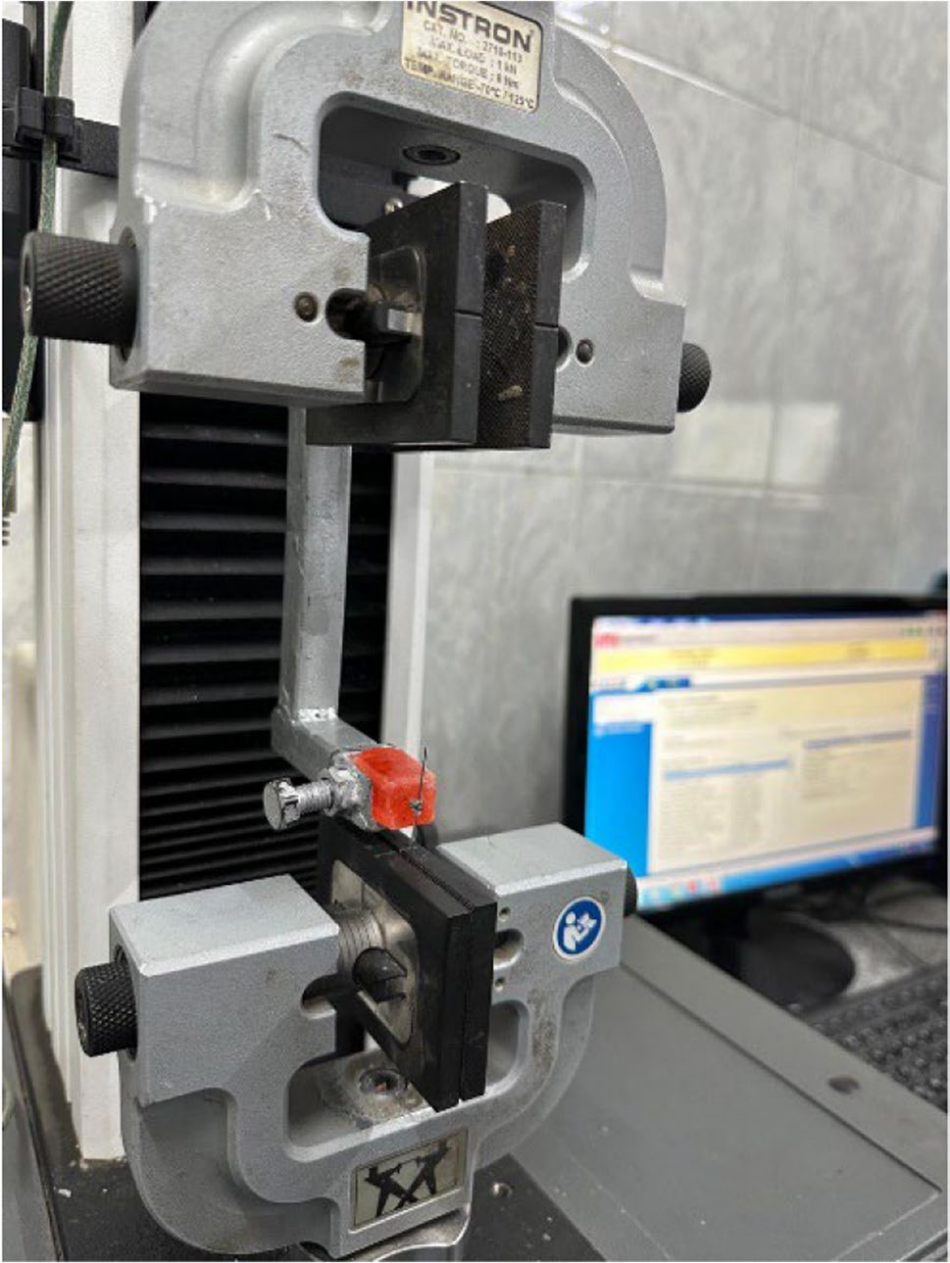
Universal testing machine (Instron 2519 − 104), Norwood, USA) with bracket fixed on the acrylic plate attached with wire sample measuring the static friction

Using Lower incisor brackets of 0.022 × 0.028 inches slot size, SS wires (0.017 × 0.025 inches), NiTi wires (0.017 × 0.025 inches) and elastic modules of the same color and brand were used for static friction evaluation. Each bracket was attached to a cylindrical acrylic block. Next, the test specimen was attached to the modified jig on the Universal Testing Machine (Instron 2519 − 104). The wire was then used to calibrate the bracket al.ignment at the jig, ensuring that it lay level in the bracket slot and protected from torsion and the tooth movement was replicated by placing the wire along the bracket. The universal testing equipment measured frictional force with a constant load of 50 N was applied during the friction testing procedure according to previous studies [25–28]. The sliding distance during friction testing was standardized to 5 mm for all samples. At a constant crosshead speed of 1 mm/min, this corresponded to a total displacement time of approximately 1 min per test. This standardized displacement ensured consistent measurement of peak static friction across all specimens [26, 29–32]. To ensure adequate bracket–wire engagement and standardized testing conditions, the bracket–wire assembly was carefully aligned to ensure that the arch wire was positioned passively within the bracket slot without angulation or torsional distortion. The wire was then moved vertically at a constant crosshead speed of 1 mm/min using a universal testing machine (Fig. 3).

**Fig. 4:**
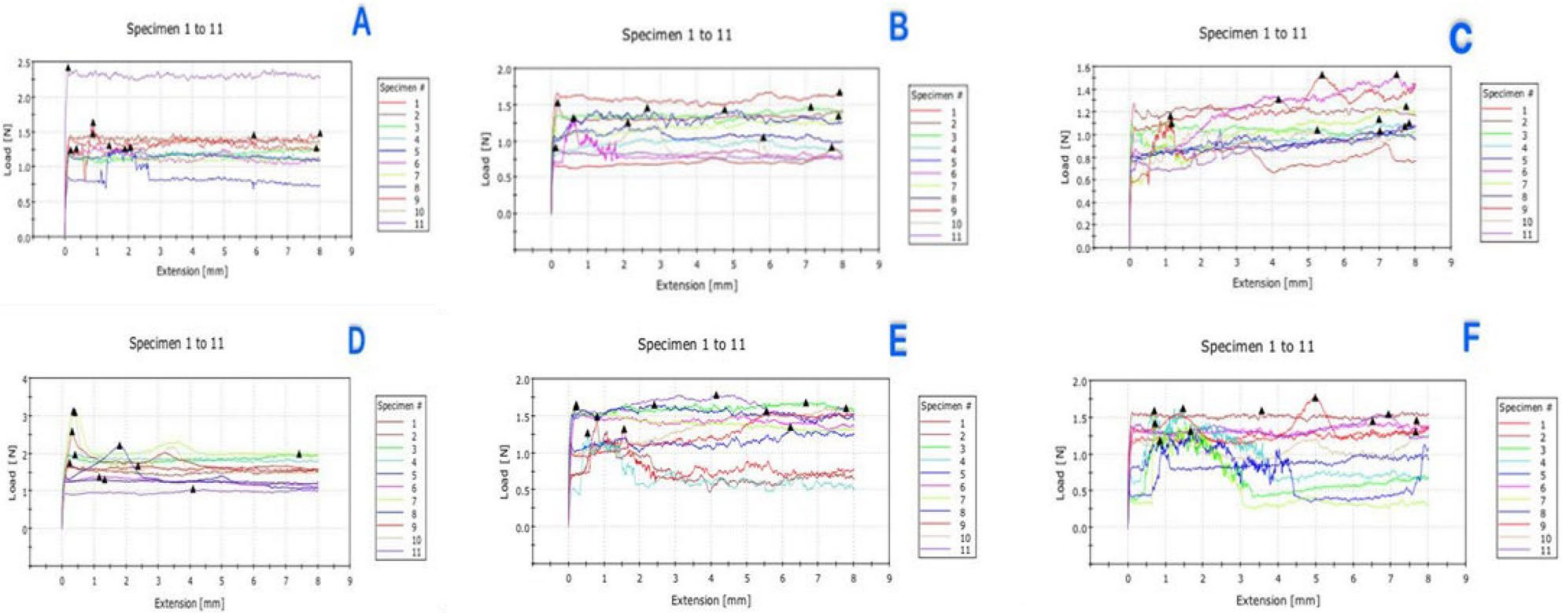
The graph of recorded results of friction as the greatest static friction for each sample, shown by the graph’s peak, was thereafter recorded automatically by the computer. **A** for the non-coated SS group (control), **B** TiO_2_NPs coated SS group, **c** Ag-NPs coated SS group, **D** for the non-coated NiTi group (control), **E** TiO_2_NPs coated NiTi group, **F** Ag-NPs coated NiTi group

To reduce wear on frictional force, each arch wire specimen was tested only once and to avoid potential surface alterations caused by repeated sliding cycles which could influence friction measurements and affect the reliability of the results. Peak static friction for each sample given by the graph, was thereafter recorded automatically by the computer (Fig. 4). Subsequently, the machine was halted to extract the old sample, and a fresh assembly of bracket and wire was installed. This study was conducted under dry conditions to prevent contamination [26].

### Surface roughness test

A profilometer (Mitutoyo 178-560-01D, Surftest SJ-21, Sakado, Japan) was used to measure surface roughness R_a_ after verifying calibration and adjusting parameters (0.5 mm/s, λc 0.08, × 5) [33]. The arch wire was attached to a plastic block. Each sample was measured three times. The statistical analysis was conducted on the mean R_a_ values in (µm). Prior to measurements, the profilometer was calibrated according to the manufacturer’s instructions to ensure the accuracy and reliability of the surface roughness readings as a scanning length of 4.0 mm was utilized, with vertical displacement captured at a precision of ± 0.01 μm. Surface roughness (Ra) was automatically calculated by the software from the recorded profilometric tracks [34, 35]. For each wire category, three independent scans were conducted on distinct samples, with results expressed as mean ± standard deviation (SD).

### Ion release test

**Fig. 5:**
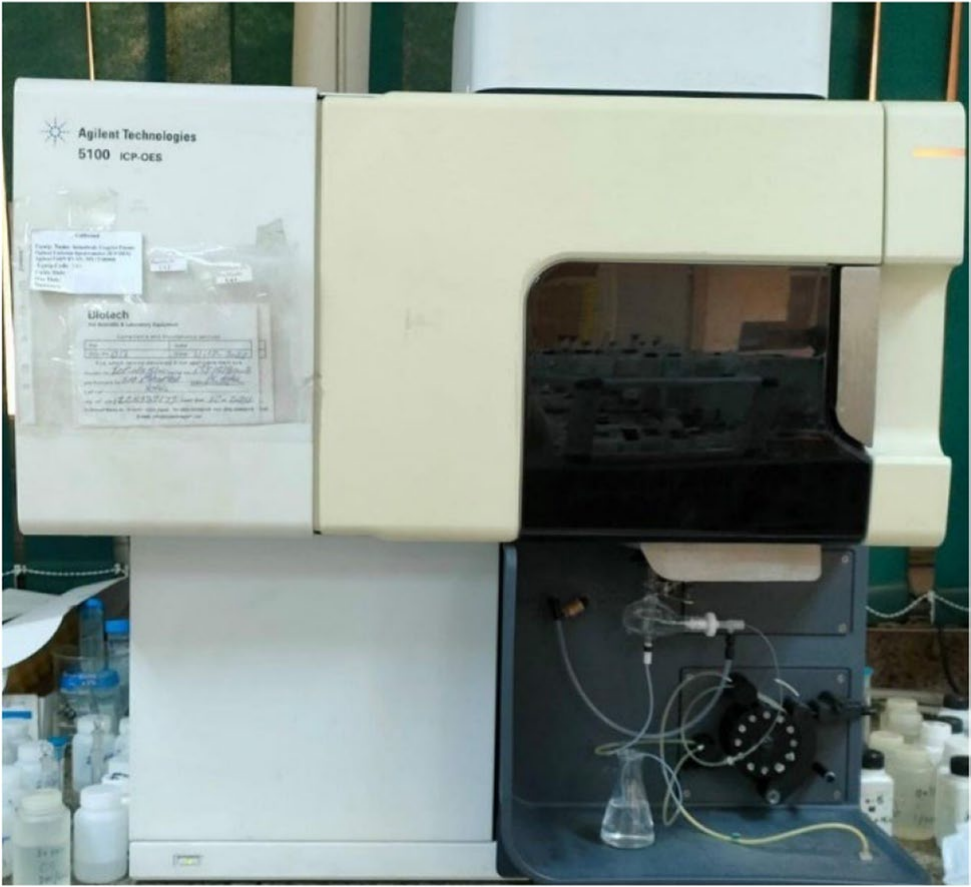
Inductively coupled plasma-optical emission spectroscopy (ICP-OES) instrument (the Agilent 5100 Synchronous Vertical Dual View, or SVDV) instrument, in conjunction with the Agilent Vapor Generation Accessory VGA 77) which used in measuring of ion release test

The ion release tests were carried out in artificial saliva environment of 6.75 pH, created at the Faculty of Pharmacy, in Mansoura in accordance with Fusayama’s composition, consisting of NaCl, KCl, Na_2_S·9H_2_O, urea (CO(NH_2_)_2_), and NaH_2_PO_4_ mimicking the physiological circumstances of the oral cavity [36]. The analysis of Ni, Ag and Ti ions release was conducted by completely immersing 35.6 mm length of uncoated and coated SS and NiTi arch wires in 10 mL of Fusayama’s artificial saliva solution within sterile polypropylene containers that were sealed during incubation. The artificial saliva solution was not refreshed during the immersion period to allow cumulative ion release measurement for a duration of 48 h, 1 week and 2 weeks. All specimens had identical wire dimensions and lengths to ensure consistent exposed surface area across all tested samples. The amount of Ni, Ag and Ti ions released by incubating 36 orthodontic arch wire samples in polypropylene containers with 10 mL of Fusayama’s artificial saliva solution were measured. With a constant temperature of 37 °C, the incubation times varied from 48 h to two weeks. Following the incubation periods, a quantitative assessment of the liberated metal ions was conducted utilizing inductively coupled plasma-optical emission spectroscopy (the Agilent 5100 Synchronous Vertical Dual View (SVDV) ICP-OES, equipped with the Agilent Vapor Generation Accessory VGA 77) (Fig. 5) The analysis was performed in the water pollution research unit of the National Research Center in Cairo, guaranteeing the reproducibility of the results. Possible sources of mistake encompassed contamination from reagents, which might introduce contaminants that influence the readings. This was alleviated by employing ultrapure reagents for the preparation of all solutions and samples [13].

### Statistical analysis test

Using IBM-SPSS software (IBM Corp. Released 2020), data was entered and evaluated. GraphPad Prism 9.5.1 for Windows and IBM SPSS Statistics for Windows, Version 27.0, Armonk, NY: IBM Corp were utilized., The normality of the quantitative data was first checked with Q-Q plots and Shapiro-Wilk’s test (*p* > .050 indicates normally distributed data). Significant outliers were checked for in the boxplots. Mean and standard deviation (SD) or standard error (SE) were used to express quantitative data. To find out if the two independent factors (coating NPs and arch wire) interact with the continuous dependent variable (surface roughness), we utilized the two-way ANOVA test. The primary effects of the arch wire and coated NPs were then reported, since the interaction effect had no statistical significance. A statistically significant difference was quantified using partial eta squared (η2). If the partial eta squared (η2) was 0.01, 0.06, or 0.14, the effect size was categorized as small, medium or large respectively. When the *p*-value was less than or equal to.050, the results were deemed statistically significant for all tests. In the ion release test, to comprehend the impact of wire type, nanoparticle type and time on the parameters under study, a three-way mixed ANOVA was conducted. Using Levene tests, we checked for variance homogeneity (*p* > .05). A two-way interaction between the wire and nanoparticle was conducted with respect to time in order to achieve a three-way interaction. At each time point, a two-way interaction between the wire and nanoparticle was conducted in order to achieve a three-way interaction. The occurrence of simple-simple main effects was reported for a simple two-way interaction when it shows statistically significant. It was from the outcomes of pairwise comparisons that simple-simple comparisons were reported for simple-simple main effects that show statistically significance. Using Greenhouse-Geisser method as sphericity was not assumed (*p* < .001) and Epsilon small (i.e., < 0.750). In addition to normality testing, independence of observations was ensured through the experimental design, Assumptions for one-way ANOVA were fulfilled as one dependent variable that was measured at the continuous level, one independent variable that was consisted of multiple independent groups, independence of observations, i.e., there was no relationship between the observations in each group of the independent variable or between the groups themselves, no significant outliers in all the groups of the independent variable, the dependent variable was approximately normally distributed for each group of the independent variable and the variance of the dependent variable was also assessed by Levene’s test of homogeneity of variances.

## Results

### Static friction test

**Fig. 6:**
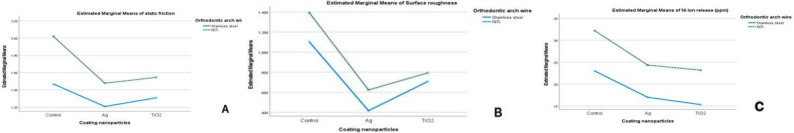
**A** Static friction in both arch wires in different nanoparticles, **B** Surface roughness in both arch wires in different nanoparticles, **C** Ni ion release in both arch wires in different nanoparticles at 48 h

There was a statistically significant main effect of the arch wire type with large effect size as the static friction was higher in NiTi wire than SS wire (*p* < .001) (Fig. 6. A). Additionally, a statistically significant main effect of the nanoparticle type with large effect size was found as the static friction was significantly higher in control than TiO_2_NPs and Ag-NPs regardless nano-coatings type (*p* < .001). The both coating types decreased the static friction nearly at the same range as Ag-NPs were very slightly more effective than TiO_2_NPs. The interaction between type of coating material and arch wire materials was insignificant (*p* = .269) with small effect size (Table 1).

**Table 1:** Static friction evaluation in coated and uncoated SS and NiTi arch wires in N

Arch wire	NPs	Mean (*N*)	SD	F (2, 60)	Sig.	Partial η^2^	95% CI
SS	Control	1.4684	0.34104	1.341	0.269	0.043	1.27–1.67
	Ag-NPs	1.2084	0.17976				1.10–1.31
	TiO_2_NPs	1.3094	0.24962				1.16–1.46
NiTi	Control	2.0202	0.68983				1.61–2.43
	Ag-NPs	1.4776	0.16809				1.38–1.58
	TiO_2_NPs	1.5451	0.16450				1.45–1.64

Main effect of arch wires on the static friction.							
Arch wire	*n*	Mean (N)	SE	F (1, 60)	Sig.	Partial η^2^	
SS	33	1.329	0.061	16.552	< 0.001	0.216	
NiTi	33	1.681	0.061				

Main effect of coating NPs on the static friction.							
NPs	*n*	Mean (N)	**SE**	F (2, 60)	Sig.	Partial η^2^	
Control	11	1.744	0.075	7.965	< 0.001	0.210	
Ag NPs	11	1.343	0.075				
TiO_2_NPs	11	1.427	0.075				

*SD*  standard deviation. *Sig*. statistical significance (*p*-value). Partial eta squared (η^2^) is a measure of effect size. F (2, 60)  F-statistic at 2 and 60 degrees of freedom. F (1, 60)  F-statistic at 1 and 60 degrees of freedom. The test of significance is two-way ANOVA

### Surface roughness test

There was a statistically significant main effect of the arch wire type with large effect size as the surface roughness was higher in NiTi wire than SS wire (*p* < .001) (Fig. 6. B). A statistically significant main effect of nanoparticle type with large effect size was found as the surface roughness was significantly higher in control > TiO_2_NPs > Ag-NPs (*p* < .001). The interaction between type of coating material and arch wire materials was insignificant (*p* = .165), with small effect size (Table 2).

**Table 2:** Surface roughness evaluation in coated and uncoated SS and NiTi arch wires in µm

Arch wire	NPs	Mean (µm)	SD	F (2, 60)	Sig.	Partial η^2^	95% CI
SS	Control	1.10182	0.169998	1.859	0.165	0.058	1.00–1.20
	Ag-NPs	0.41712	0.098426				0.36–0.47
	TiO_2_NPs	0.70688	0.170006				0.61–0.81
NiTi	Control	1.39233	0.323612				1.20–1.58
	Ag-NPs	0.62321	0.057300				0.59–0.66
	TiO_2_NPs	0.79321	0.106825				0.73–0.86

Main effect of arch wires on the surface roughness.							
Arch wire	*n*	Mean (µm)	SE	F (1, 60)	Sig.	Partial η^2^	
SS	33	0.742	0.031	19.999	< 0.001	0.250	
NiTi	33	0.936	0.031				

Main effect of coating NPs on the surface roughness.							
NPs	*n*	Mean (µm)	SE	F (2, 60)	Sig.	Partial η^2^	
Control	11	1.247	0.038	97.492	< 0.001	0.765	
Ag-NPs	11	0.520	0.038				
TiO_2_NPs	11	0.750	0.038				

*SD*  standard deviation. *Sig*. statistical significance (*p*-value). Partial eta squared (η^2^) is a measure of effect size. F (2, 60) = F-statistic at 2 and 60 degrees of freedom. F (1, 60) = F-statistic at 1 and 60 degrees of freedom. The test of significance is two-way ANOVA

### Ion release test

#### Ni ion release

In comparison of time interval after 48 h with time interval after 1 week and after 2 weeks there was a statistically significant three-way interaction between orthodontic arch wire type, coating nanoparticle type, and time on Ni ion release [F (2.9, 43) = 4.463, partial eta squared ( η^2^) = 0.229, *p* = .009] using Greenhouse-Geisser method as sphericity was not assumed (*p* < .001) and Epsilon was 0.716 (i.e., < 0.750). Therefore, simple two-way interaction procedures were performed. As statistical significance of a simple two-way interaction was accepted at a Bonferroni-adjusted alpha level of 0.0167 (0.05/3 (number of simple two-way interactions).

After 48 h there was a statistically significant main effect of the arch wire type with small effect size (*p* < .001) being significantly higher in NiTi wire than SS wire (Table 5). Additionally, there was a significant difference in Ni ion release between nanoparticle types (*p* < .001) as the release was higher in control > Ag-NPs > TiO_2_NPs with large effect size (Fig. 6. C). Similarly, after 1 week, the main effect of wire type was statistically significant (*p* < .001) as it was higher in the NiTi group than the SS group. Also, the main effect of nanoparticle type was statistically significant (*p* < .001) revealed a higher Ni ion release in control group > Ag-NPs group > TiO_2_NPs group. There was a statistically insignificant simple two-way interaction effect of orthodontic arch wire type and coating nanoparticle types on Ni ion release at 48 h (*p* = .872) and at 1 week (*p* = .398). In contrast after 2 weeks there was a statistically significant simple two-way interaction effect of orthodontic arch wire type and coating nanoparticle types on Ni ion release (*p* = .021) so, there were statistically significant simple-simple main effects after 2 weeks between the nanoparticle groups in SS wire (*p* < .001 for all), and between TiO_2_NPs group vs. both control group and Ag-NPs group in NiTi wire (*p* < .001, and 0.038, respectively), but not significant between control group vs. Ag-NPs group (*p* = .322). Also, there was a statistically significant difference between the two wires in Ag-NPs and TiO_2_NPs groups (*p* < .001 for both) (Table 3).

**Table 3:** The interactions between ion release of Ni ions (ppm) in SS and NiTi arch wires over time interval of 48 h, 1 week and 2 weeks

The relation of ion release of Ni with time intervals of 48 h, 1 week and 2 weeks.						
Ion release		Time-point	Mean difference (ppm)	F (1, 34)	Sig.	Partial η^2^
Ni		At 48 h	-0.081	18.799	< 0.001	0.356
		At 1-week	-0.072	11.143	0.002	0.247
		At 2-weeks	-0.081	11.604	< 0.001	0.286

Pairwise comparisons of Ni ion release in each of the control, Ag NPs and TiO_2_NPs coated wires groups at 2 weeks.						
		Mean difference (ppm)	SE	Sig.	95% CI	
					Lower	Upper
Control		− 0.035	0.020	0.092	− 0.076	0.006
Ag-NPs		− 0.088	0.020	< 0.001	− 0.129	− 0.047
TiO_2_NPs		− 0.118	0.020	< 0.001	− 0.159	− 0.077

Pairwise comparisons of Ni ion release between the control, Ag-NPs and TiO_2_NPs groups in each of the SS and NiTi wires at 2 weeks.						
		Mean difference (ppm)	SE	Sig.	95% CI	
					Lower	Upper
Control vs. Ag-NPs	SS	0.087	0.020	< 0.001	0.036	0.138
	NiTi	0.033	0.020	0.322	− 0.018	0.084
Control vs. TiO_2_NPs	SS	0.170	0.020	< 0.001	0.119	0.221
	NiTi	0.087	0.020	< 0.001	0.036	0.138
Ag-NPs vs. TiO_2_NPs	SS	0.083	0.020	0.001	0.032	0.134
	NiTi	0.053	0.020	0.038	0.002	0.104

Sig. statistical significance (*p*-value). CI   confidence interval. The test of significance is three-way mixed ANOVA.

mean difference   Ni ion release in stainless steel *minus* NiTi. SE   standard error, Sig.  statistical significance (*p*-value). CI   confidence interval

mean difference   Ni ion release in 1st subgroup minus 2nd subgroup.SE   standard error. Sig.  statistical significance (*p*-value). CI   confidence interval

#### Ag ion release

After 48 h there was a statistically insignificant main effect of arch wire type with small effect size (*p* = .386) (Table 5). There was a significant difference in Ag ion release between nanoparticle type (*p* < .001), being higher in Ag-NPs group > TiO_2_NPs group and control group with large effect size. The interaction between the type of coating materials and arch wire materials was insignificant (*p* = .471), with small effect size. Ag ion release in both types of wire was in the safe and biocompatible concerns.

Comparison of time interval after 48 h, 1 week and 2 weeks, revealed no statistically significant three-way interaction between orthodontic arch wire type, coating NPs type and time on Ag Ion release [F (2.7, 40.9) = 28.248, partial eta-squared ( η^2^) = 0.021, *p* = .795] using Greenhouse-Geisser method as sphericity was not assumed (*p* < .001) and Epsilon was 0.681 (i.e., < 0.750). As a result, regarding orthodontic arch wire types and time, there was no statistically significant two-way interaction effect [F (1.2, 42.2) = 0.210, partial eta-squared (η^2^) = 0.006, *p* = .702] using Greenhouse-Geisser method as sphericity was not assumed (*p* < .001) and Epsilon was 0.620 (i.e., < 0.750) but, regarding coating nanoparticle types and time, there was a statistically significant two-way interaction effect [F (2.7, 45.2) = 10.888, partial eta-squared (η^2^) = 0.398, *p* < .001] using Greenhouse-Geisser method as sphericity was not assumed (*p* < .001) and Epsilon was 0.684 (i.e., < 0.750). Accordingly, pairwise comparisons of Ag ion release between NPs groups are reported (Table 4).

**Table 4:** The interactions between ion release of Ag and Ti ions (ppm) in SS and NiTi arch wires over time interval of 48 h, 1 week and 2 weeks

The interactions between ion release of Ag and Ti ions (ppm) in SS and NiTi arch wires over time interval of 48 h, 1 week and 2 weeks.						
Ion release		Time-point	Mean difference SS – NiTi (ppm)	F (1, 34)	Sig.	
Ag		At 48 h	0.004	0.055	0.816	
		At 1-week	0.005	0.074	0.788	
		At 2-weeks	0.006	0.102	0.752	
Ti		At 48 h	-0.031	1.150	0.291	
		At 1-week	-0.058	3.867	0.057	
		At 2-weeks	-0.091	8.582	0.006	

Pairwise comparisons of Ag and Ti Ion release (ppm) for nanoparticles and time over all time intervals.						
Subgroup		Mean difference (ppm)	SE	Sig.	95% CI	
					Lower	Upper
Control vs. Ag-NPs	Ag	− 0.110	0.004	< 0.001	− 0.119	− 0.100
	Ti	0.010	0.018	1.000	− 0.034	0.055
Control vs. TiO_2_NPs	Ag	4.626E-18	0.004	1.000	− 0.010	0.010
	Ti	− 0.168	0.018	< 0.001	− 0.213	− 0.124
Ag-NPs vs. TiO_2_NPs	Ag	0.110	0.004	< 0.001	0.100	0.119
	Ti	− 0.179	0.018	< 0.001	− 0.223	− 0.134

*mean difference*  Ni ion release in stainless steel *minus* NiTi. *SE*  standard error. *Sig*. statistical significance (*p*-value). *CI*  confidence interval

#### Ti ion release

After 48 h there was a statistically significant main effect of arch wire type with large effect size. The Ti ion release was statistically significantly higher in NiTi wire than SS wire (*p* = .018) (Table 5). There was a significant difference in Ti ion release between nanoparticle types (*p* < .001) as the release was higher in TiO_2_NPs group > Ag-NPs group and control group with large effect size. The interaction between type of coating materials and arch wire materials was insignificant (*p* = .203), with medium effect size. Similarly, Ti ion release in both types of arch wire was in the safe and biocompatible concerns. Comparison of time intervals after 48 h, 1 week and 2 weeks showed no statistically significant three-way interaction between orthodontic arch wire type, coating nanoparticle type, and time on Ti ion release [F (2.9, 43.5) = 2.476, partial eta-squared (η^2^) = 0.142, *p* = .076] using Greenhouse-Geisser method as sphericity was not assumed (*p* = .001) and Epsilon was 0.725 (i.e., < 0.750). As a result, regarding orthodontic arch wire type and time, there was a statistically significant two-way interaction effect [F (1.3, 44) = 44.007, partial eta-squared (η^2^) = 0.564, *p* < .001] using Greenhouse-Geisser method as sphericity was not assumed (*p* < .001) and Epsilon was 0.648 (i.e., < 0.750). In contrast regarding coating NPs and time, there was no statistically significant two-way interaction effect [F (2.3, 37.6) = 2.086, partial eta-squared (η^2^) = 0.112, *P*=.133] using Greenhouse-Geisser method as sphericity was not assumed (*p* < .001) and Epsilon was 0.570 (i.e., < 0.750). So, the main effect of coating NPs type was reported which revealed a statistically significant difference between the three coating NPs [F (2, 33) = 64.944, partial eta-squared (η^2^) = 0.797, *p* < .001] as shown in the pairwise comparisons between the NPs on Ti ion release showing a statistically significant higher Ti Ion release in TiO_2_NPs than both control group and Ag-NPs group (Table 4).

**Table 5:** Main effect of SS and NiTi arch wires on Ni, Ag and Ti and ion release after 48 h in (ppm)

Ion release	Arch wire	*n*	Mean (ppm)	SE	F (2, 30)	Sig.	Partial η^2^
1. Ni ion release	SS	18	0.184	0.010	30.193	< .001	0.502
	NiTi	18	0.266	0.010			
2.Ag ion release	SS	18	0.035	0.003	0.773	0.386	0.025
	NiTi	18	0.031	0.003			
3. Ti ion release	SS	18	0.046	0.009	6.272	0.018	0.173
	NiTi	18	0.077	0.009			

*Sig*. statistical significance (*p*-value). Partial eta squared (η^2^) is a measure of effect size. F (2, 60) = F-statistic at 2 and 60 degrees of freedom. The test of significance is two-way ANOVA

## Discussion

The impact of this study was to evaluate applied recent nanotechnology in orthodontics regarding the static friction, surface roughness and ion release of Ni, Ag and Ti of Ag-NPs and TiO_2_NPs coatings on NiTi and SS arch wires. Electron microscopy scanning research was performed on the coated orthodontic arch wires to evaluate the coating’s adherence to the wire [37] Although SEM imaging confirmed the presence of the nano-coatings on the arch wire surfaces, quantitative measurement of coating thickness and uniformity using techniques such as atomic force microscopy (AFM) or cross-sectional SEM was not performed [10, 23, 24]. Orthodontic wire coatings can affect the mechanical properties of the arch wire as surface roughness, frictional qualities, thickness and corrosiveness of the wires’ surfaces [38]. Ag-NPs are employed in many biological applications due to their reduced toxicity in comparison to their larger counterparts [39]. The TiO_2_NPs demonstrate satisfactory hardness and substantial corrosion resistance. Additionally, they are non-toxic and chemically inert [40]. NPs or nanocomposite materials can be applied to arch wires by sol-gel method which was adopted for this investigation due to its homogeneity and high integrity [41]. Rectangular SS and NiTi arch wires measuring 0.017 × 0.025 inches were used in this investigation. The observed statistical effect sizes suggest that for example nanoparticle coatings may have a clinically meaningful influence on frictional behavior between orthodontic brackets and arch wires. Reduced friction may facilitate more efficient sliding mechanics during orthodontic treatment and potentially improve treatment efficiency. Likely, smoother wire surfaces may reduce plaque accumulation and bacterial adhesion, while lower ion release may contribute to improved biocompatibility [42, 43].

Static friction denotes that “the minimum force necessary to commence orthodontic tooth movement when the two surfaces are in a static relationship” [44] Accordingly, friction may reduce the force used by the fixed device by more than 60% of the orthodontic force applied to achieve orthodontic tooth movement [45]. Elastomeric ligatures are applied as they are fixed all around the arch wires and bracket edges with less time for application compared to SS ligatures [46]. Furthermore, only elastomeric ligatures were used for bracket–wire ligation. Different ligation methods such as SS ligatures or self-ligating brackets, may produce different frictional characteristics [47, 48]. The acrylic plate was created and the test specimen was attached to the modified jig on the Universal Testing Machine. It is necessary to load the sliding test with 50 newtons to keep the tension in the wire and make it harder to slide [44, 45]. It should be noted that the friction test was conducted under dry laboratory conditions to standardize measurements. However, the oral environment contains saliva that may act as a lubricant and influence frictional behavior between brackets and arch wires. Therefore, the present results should be interpreted with caution when extrapolating to clinical conditions [19, 49–51]. The experimental design was standardized to reduce variability and allow direct comparison between nano-coating types. Friction testing was performed using lower incisor brackets to standardize the experimental model [52, 53]. The results of static friction in this study revealed a significant reduction in static friction in both Ag-NPs and TiO_2_NPs coated arch wires compared with uncoated wires. Both of nano-coatings decreased the static friction nearly at the same range in both arch wire types but Ag-NPs decreased the static friction slightly more than TiO_2_NPs with SS arch wires showing greater improvement than NiTi wires. Frictional resistance can be increased by nanotechnology, enabling the delivery of a more gradual and even force [19]. These findings are strongly supported by the systematic review conducted by Indumathi et al. [54] who concluded that NP coatings particularly both Ag-NPs and TiO_2_NPs coatings consistently reduce friction at the bracket wire interface. The authors emphasized that SS wires respond more favorably to surface modification, which aligns with the present results. Kielan-Grabowska et al. [22] similarly reported that Ag-NPs and TiO_2_NPs coatings applied using the sol-gel technique significantly reduced frictional resistance of SS arch wires, further validating the coating method and materials used in the current study. Hemanth et al. [19] also observed a significant decrease in friction in nano-coated arch wires compared with uncoated controls. Conversely, Usui et al. [55] further demonstrated that certain coatings can increase friction due to excessive coating thickness or uneven deposition. These contradictory findings suggest that the effectiveness of nano-coatings is highly dependent on coating uniformity, thickness, and nanoparticle dispersion, which may explain discrepancies among studies.

Similarly, the surface roughness of the coated and uncoated arch wires was measured by using profilometric method. The findings are frequently displayed as root mean square (RMS) values [33]. Only the Ra parameter was used to evaluate surface roughness in this study, as it is the most commonly reported metric in orthodontic materials research [48, 56, 57]. The present study demonstrated that nano-coated arch wires exhibited significantly lower surface roughness compared with uncoated wires, with Ag-NPs coated SS wires showing the smoothest surfaces. This observation is consistent with the findings of Amini et al. [33]. who reported that NiTi wires possess inherently higher surface roughness than SS wires due to differences in manufacturing processes. Yu et al. [35] also confirmed that surface roughness varies significantly among orthodontic wire materials, with NiTi wires displaying rougher topographies. Kielan-Grabowska et al. [22] reported a marked reduction in surface roughness following Ag-NPs and TiO_2_NPs coating of SS arch wires, supporting the effectiveness of sol-gel nano-coatings in smoothing surface irregularities. In contrast, Redlich et al. [58] reported that nanoparticle impregnated coatings can increase surface roughness if particle agglomeration occurs or coating parameters are not optimized.

The coated groups have a significant decrease in surface roughness rather than uncoated group which represents the direct relation between friction and roughness. By increasing the area of contact between the bracket and the wire, elevated surface roughness can increase frictional forces [59]. Nanomaterials can enhance the sliding mechanics between the arch wire and brackets by many techniques, including better surface smoothness, lubrication, changed texturing, and precise manufacturing, hence minimizing binding and friction. These enhancements have been facilitated by progress in nano-coatings and nanocomposite materials [60].

Metal ions, primarily Ni and Cr, from fixed orthodontic devices can cause allergic reactions [61]. Corrosion refers to electrochemical reactions that result in the degradation of a metal’s surface by ion release producing corrosive variables which are influenced by the structure of the metal, while external effects are based upon biological environments (e.g., medium composition, strain, illumination, pH, temperature) [61]. NiTi alloys, act like SS which exhibit a propensity to corrode in solutions containing chlorine and fluorine [62]. Certain surface coatings can improve corrosion resistance; nevertheless, these coatings may deteriorate and delaminate over time consequently, the coating of fixed appliances must exhibit resistance to corrosion to enhance durability [49]. In the present study we used ICP-OES, to measure ion release of Ni, Ag and Ti ions after immersing the NiTi and SS arch wires (uncoated and nano-coated) in 10 mL of Fusayama’s artificial saliva solution in a sterilized tube for periods after 48 h, 1 week and 2 weeks. Although artificial saliva was used to simulate the oral environment, its composition does not fully replicate natural saliva, which contains enzymes, proteins and microbial components that may influence corrosion and ion release behavior [63–66]. The ion release test was performed in static artificial saliva at a constant temperature of 37 °C. However, dynamic oral conditions such as pH cycling and temperature fluctuations were not simulated [65, 67].

The results of this study demonstrated a significant reduction in Ni ion release in nano-coated arch wires, particularly in SS wires coated with TiO_2_NPs. These findings are consistent with Mikulewicz et al. [13] who reported that surface-modified orthodontic wires released significantly lower levels of Ni ions compared with uncoated wires. Chaturvedi et al. [49] also demonstrated that TiO_2_NPs coatings act as effective electrochemical barriers, reducing Ni ion release by limiting charge transfer and metal–saliva interaction. Bacela et al. [38] emphasized that oxide-based nano-coatings enhance corrosion resistance due to their low electrical conductivity and chemical stability. However, Stoyanova-Ivanova et al. [61] reported that Ni ion release may persist over time due to coating degradation and micro-crack formation, particularly in NiTi alloys exposed to mechanical stress. These findings explain the higher Ni ion release observed in NiTi wires compared with SS wires in the present study.

In the current study, Ag and Ti ions release levels remained within biologically safe limits throughout the experimental period. These results agree with Yin et al. [68] who reported excellent biocompatibility of Ag-NPs in dental applications. Oves et al. [69] demonstrated that Ag-NPs used in biological and medical applications at very low ion concentrations with minimal cytotoxicity. Similarly, Baranowska-Wójcik et al. [70] reported that TiO_2_NPs show minimal toxicity when used at concentrations relevant to dental applications. Mohammadi et al. [40] further confirmed that TiO_2_NPs are chemically inert and suitable for long-term use in dentistry. Azizi et al. [67] found that both round and rectangular NiTi wires reach their maximum release of metal ions at day 10, with rectangular wires showing higher concentrations due to their larger surface area. Despite this peak, they reported that the total ion release remains well below toxic thresholds, though it remains clinically relevant for patients with specific metal hypersensitivities.

### Limitations

Despite the valuable findings of the present study, several limitations should be acknowledged. The present study evaluated the release of Ag and Ti ions to assess the potential biocompatibility of the NPs coatings and the direct detachment of intact NPs. The immersion period in the present study was limited to two weeks, which reflects short-term ion release behavior. Longer evaluation periods may be necessary to better simulate the long-term clinical conditions of orthodontic treatment. The present study focused on the common ions associated with both tested orthodontic wires and the ions of the nano coatings to evaluate the toxicity but did not evaluate Cr ion release only from SS wires, which may also contribute to corrosion-related biological effects [71].

Additionally, the wear resistance and long-term durability of the nano-coatings during sliding mechanics were not evaluated in this study [9, 10, 23, 72]. Furthermore, complex intraoral factors such as mastication forces, biofilm accumulation, enzymatic activity and mechanical stresses were not simulated in the present in vitro study which may influence the long-term behavior of coated orthodontic wires [13, 65, 73]. Although standardized coating procedures were used to deposit the NPs on the orthodontic arch wires, the exact thickness of the coatings was not quantitatively measured. Coating thickness may influence both frictional resistance and ion release behavior; therefore, future investigations should include quantitative characterization methods such as cross-sectional SEM or AFM to provide a more comprehensive evaluation of coating morphology [10, 23, 24]. In the present study, only static friction was evaluated because it represents the initial resistance that must be overcome to initiate tooth movement during orthodontic sliding mechanics. However, kinetic friction, which occurs during continuous sliding, was not assessed and may also influence the overall frictional behavior of orthodontic appliances [74, 75]. In addition, only one type of orthodontic bracket system was used in this investigation to standardize the experimental setup. Since different bracket designs may influence frictional resistance and the generalizability of the findings [76, 77]. This study was conducted under dry laboratory conditions to standardize measurements and minimize experimental variability. However, it should be emphasized that standardization is not limited to dry testing conditions. Controlled wet environments, such as artificial saliva setups, can also provide reproducible conditions while better simulating the clinical oral environment. Saliva may act as a lubricant and influence frictional behavior between brackets and arch wires. Therefore, the present findings should be interpreted with caution when extrapolated to in vivo conditions [26, 47, 78].Surface roughness measurements were performed only after the coating process and not following friction testing; therefore, potential changes in surface morphology due to wear during sliding mechanics were not evaluated. The present study evaluated the working arch wire dimension (0.017 × 0.025 inches). Since different wire sizes may influence frictional behavior during orthodontic sliding mechanics well as that wire dimensions were used in brackets of 0.022 × 0.028 inches slot size [48, 75].

The experiments were conducted under controlled in vitro conditions that do not fully replicate the complex oral environment. Factors such as saliva dynamics, pH fluctuations, mastication forces, biofilm formation, and enzymatic activity were not simulated. In addition, coating thickness and uniformity were not quantitatively measured, and the wear resistance of the coatings during sliding mechanics was not evaluated. Furthermore, only one bracket type, ligation method, and wire dimension were tested. Therefore, further in vivo studies and long-term investigations are recommended to better evaluate the clinical performance of nanoparticle-coated orthodontic arch wires.

### Recommendations

Future studies should include quantitative thickness measurements using advanced techniques such as cross-sectional SEM or atomic force microscopy (AFM) to better characterize the coating properties and should include AFM and SEM analyses to better characterize coating morphology. Wear resistance of the coatings during sliding mechanics should be investigated in future research. The present study focused primarily on the effect of NPs coatings on the frictional behavior of orthodontic arch wires. Therefore, potential surface alterations or wear of orthodontic brackets after friction testing were not evaluated. Future investigations should assess bracket surface wear following sliding mechanics to provide a more comprehensive understanding of the bracket–wire interaction. Frictional behavior may vary in different regions of the dental arch, particularly in posterior teeth, and should be evaluated in future studies. Future studies should include Cr analysis for a more comprehensive evaluation.

## Conclusion

There was a complete rejection of the null hypothesis which decade that coating SS and NiTi orthodontic arch wires with Ag-NPs and TiO_2_NPs would not considerably increase static friction, surface roughness, corrosion resistance. Within the limitations of this in vitro study, Ag-NPs and TiO_2_NPs coatings significantly improved the performance of SS and NiTi orthodontic arch wires. Both nano-coatings reduced static friction and surface roughness compared with uncoated wires, with SS arch wires showing greater responsiveness to surface modification. Ag-NPs produced the greatest reduction in surface roughness, whereas TiO_2_NPs NPs demonstrated superior corrosion resistance by significantly decreasing Ni ion release, particularly in SS arch wires over one week and two weeks Importantly, Ag and Ti ion release remained within biologically safe limits throughout the experimental period. These findings indicate that nano-coated orthodontic arch wires can enhance sliding mechanics and biocompatibility. NPs coatings can reduce frictional resistance by improving surface smoothness and modifying the surface characteristics of orthodontic wires.

## Supplementary Information

Supplementary Material 1.

## Data Availability

All datasets used and analyzed during the current study are available from the corresponding author on reasonable request.

## Abbreviations

NPs: Nanoparticles
Ag: Silver
Ti: Titanium
Ni: Nickel
TiO₂: Titanium dioxide
Ag-NPs: Silver nanoparticles
TiO₂NPs: Titanium dioxide nanoparticles
TEA: Triethylamine
TTiP: Titanium isopropoxide
NiTi: Nickel-Titanium alloy
SS: Stainless-steel
SEM: Scanning Electron Microscope
AFM: atomic force microscopy
ANOVA: Analysis of variance
CI: Confidence interval
F: F-statistic
SD: Standard deviation
SE: Standard error
IBM-SPSS: International Business Machines Statistical Package for the Social Sciences
LV: Low vacuum
Ra: Arithmetic mean surface roughness
pH: Potential of hydrogen
RMS: Root mean square
ICP-OES: Inductively Coupled Plasma–Optical Emission Spectroscopy
