# Supplementary material for: Evaluation of static friction, surface roughness and ion release of stainless steel and nickel-titanium orthodontic arch wires coated with titanium dioxide nanoparticles and silver nanoparticles: in vitro study
Source: BMC Oral Health. 2026 May 15;26:894. doi: 10.1186/s12903-026-08518-w (PMC13200385; doi:10.1186/s12903-026-08518-w)
Supplement: Supplementary file 1 — Supplementary Material 1. [file 12903_2026_8518_MOESM1_ESM.docx]

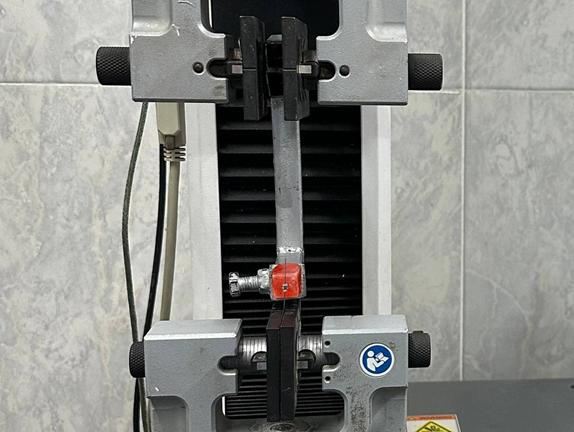


Figure. 1. Universal testing machine (Instron 2519-104), Norwood, USA) with bracket fixed on the acrylic plate attached with wire sample measuring the static friction.

**Table 1: Mean and standard deviation of Ni, Ag and Ti ion release (ppm) on time interval of (48 hours, 1 week & 2 weeks)**

| ion release | Arch wire | Nanoparticle | Mean ± SD (48h) | Mean ± SD (1w) | Mean ± SD (2w) |
| --- | --- | --- | --- | --- | --- |
| Ni ion release | **SS** | **Control** | .2300 ± .03633 | .3500 ± .03578 | .4533 ± .03011 |
|  |  | **Ag** | .1700 ± .03162 | .3067 ± .01751 | .3667 ± .02338 |
|  |  | **TiO_2_** | .1533 ± .03011 | .2083 ± .02317 | .2833 ± .02422 |
|  | **NiTi** | **Control** | .3217 ± .06735 | .4100 ± .05329 | .4883 ± .02858 |
|  |  | **Ag** | .2433 ± .04320 | .3650 ± .02881 | .4550 ± .05282 |
|  |  | **TiO_2_** | .2317 ± .04622 | .3050 ± .05577 | .4017 ± .04021 |
| Ag ion release | **SS** | **Control** | .0000 ± .00000 | .0000 ± .00000 | .0000 ± .00000 |
|  |  | **Ag** | .1050 ± .02665 | .1200 ± .01414 | .1267 ± .01211 |
|  |  | **TiO_2_** | .0000 ± .00000 | .0000 ± .00000 | .0000 ± .00000 |
|  | **NiTi** | **Control** | .0000 ± .00000 | .0000 ± .00000 | .0000 ± .00000 |
|  |  | **Ag** | .0933 ± .01862 | .1050 ± .01643 | .1083 ± .00983 |
|  |  | **TiO_2_** | .0000 ± .00000 | .0000 ± .00000 | .0000 ± .00000 |
| Ti ion release | **SS** | **Control** | .0000 ± .00000 | .0000 ± .00000 | .0000 ± .00000 |
|  |  | **Ag** | .0000 ± .00000 | .0000 ± .00000 | .0000 ± .00000 |
|  |  | **TiO_2_** | .1383 ± .02927 | .1517 ± .01329 | .1583 ± .01169 |
|  | **NiTi** | **Control** | .0150 ± .00548 | .0550 ± .01643 | .0933 ± .00816 |
|  |  | **Ag** | .0150 ± .00548 | .0333 ± .01033 | .0533 ± .01033 |
|  |  | **TiO_2_** | .2017 ± .08612 | .2383 ± .06676 | .2850 ± .04506 |
